# Tetraventricular Hydrocephalus Due to Idiopathic Fourth Ventricle Outlet Obstruction: A Case Report and Literature Review

**DOI:** 10.1055/a-2713-5787

**Published:** 2025-10-13

**Authors:** Guramritpal Singh

**Affiliations:** 1 Department of Neurosurgery, Shree Ram Neuro Centre, Jalandhar, Punjab, India

**Keywords:** idiopathic, fourth ventricle outlet obstruction, hydrocephalus, Luschka, Magendie, endoscopic third ventriculostomy, foramina

## Abstract

**Introduction:**

Tetraventricular hydrocephalus happens due to the fourth ventricle outlet obstruction. Idiopathic fourth ventricle outlet obstruction (IFVOO) is a condition where no clear-cut etiology for fourth ventricle outlet obstruction can be found. The etiopathogenesis of IFVOO is unclear. There is no clear-cut consensus regarding the treatment practices for its management. These cases present a diagnostic dilemma to the treating neurosurgeon and are thus often managed inappropriately. This study aims to review the existing literature regarding this condition, illustrating with a case from our hospital.

**Case Details:**

We present a case of a 50-year-old female who presented to us with the chief complaints of headache, difficulty in walking, with an inability to balance while standing and walking, diplopia, and three episodes of loss of consciousness for 6 months. A brain MRI was done, which was suggestive of dilatation of all ventricles with obstruction at the foramina of Luschka and Magendie. She underwent a right-sided, medium-pressure ventriculoperitoneal shunt at our hospital. Postsurgery, there was immediate improvement in her symptoms.

**Conclusion:**

IFVOO is a rare cause of tetraventricular hydrocephalus with an unknown cause. Endoscopic third ventriculostomy (ETV) appears to have a higher risk of failure in such cases. Fenestration procedures after craniotomy and shunt procedures are still effective in their management. ETV is still an alternative to the above-mentioned procedures. To confirm these conclusions, larger studies involving multiple hospitals and institutes are required.

## Introduction

Hydrocephalus is caused due to the accumulation of excess cerebrospinal fluid (CSF) in the ventricular system of the brain. In the event of obstruction of the fourth ventricle outlet (foramina of Luschka and Magendie), dilatation of all four ventricles occurs (tetraventricular hydrocephalus). Etiology includes intracranial bleed, intracranial infection (bacterial, tubercular, cysticercal meningitis) Dandy–Walker malformation, Arnold–Chiari malformation, Basilar invagination and other craniovertebral junction abnormalities, and tuberous sclerosis. 1 In rare cases, no causative factor can be identified that is causing fourth ventricle outlet obstruction (FVOO). Such a condition is called idiopathic fourth ventricle outlet obstruction (IFVOO). Congenital occlusion of the foramina of Luschka and Magendie is known to occur; however, the etiology is unknown. 2 3 As such cases are rare, only a handful of them are described in the literature. In this paper, the author presents his own experience with IFVOO and reviews the relevant literature.

## Case Details

### Clinical Presentation

**Fig. 1:**
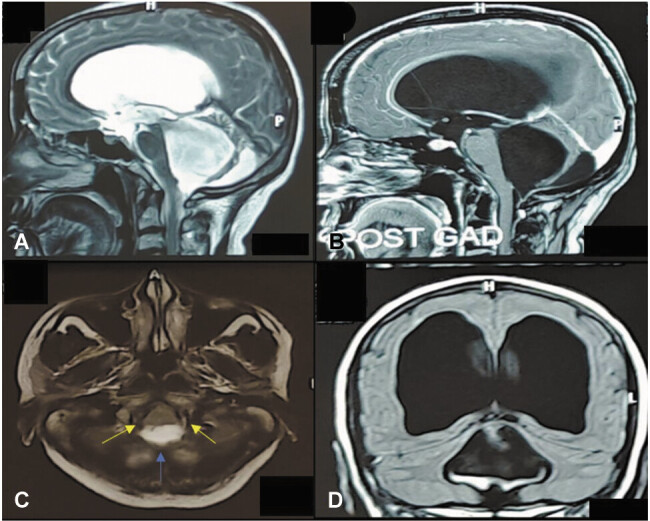
Brain MRI ( **A** - **D** ) showing tetraventricular hydrocephalus with obstruction of foramina of Luschka (yellow arrow) and Magendie (blue arrow).

A 50-year-old female presented to our hospital with the chief complaints of headache, difficulty in walking, imbalance while standing and walking, diplopia, vertigo, and three to four episodes of loss of consciousness for the past 6 months. Initially, the patient had taken medications from a local physician for the same, but no symptomatic relief occurred. On examination, the patient had bilateral modified Friesen grade III papilledema, positive bilateral cerebellar signs. A brain MRI ( Fig. 1 ) was done, which was suggestive of tetraventricular hydrocephalus with obstruction of the foramina of Luschka and Magendie. The preoperative radiological diagnosis was IFVOO. Laboratory findings were normal.

### Surgery

The patient underwent a right-sided medium-pressure ventriculoperitoneal shunt at the Keen's point. CSF came under high pressure, in a jet-like fashion, at the rate of 80 to 90 drops/minute.

### Postoperative Course and Follow-Up

On postoperative day 1, the patient reported significant improvement in gait ataxia, vertigo, and diplopia. The rest of the postoperative course in the hospital was uneventful. At the 3-month follow-up, the patient had complete resolution of the presenting complaints.

## Literature Review

A comprehensive review of the literature was undertaken to examine the management of IFVOO. We searched on the PubMed database using the terms “FVOO,” “tetraventriculomegaly,” and “hydrocephalus.” Inclusion criteria: Cases of tetraventricular hydrocephalus that did not have any associated congenital malformation or any other secondary cause, such as intracranial bleed, intracranial infection, or tuberous sclerosis. Excluding criteria: Cases of communicating hydrocephalus; secondary causes such as congenital malformation, intracranial bleed, intracranial infection, tuberous sclerosis; case reports that provided incomplete information regarding clinical presentation, radiological findings, management protocols, and functional outcomes. About 24 articles (a total of 63 cases of IFVOO) fulfilled our criteria and were included in this study, besides my own case ( Table 1 ).

**Table 1:** Literature review of Idiopathic fourth ventricle obstruction cases

S.No	Study, year	Age/Sex (M/F)	Number of patients	Clinical presentation	Surgery	Outcome	Resurgery	Mean follow-up
1	Coleman and Troland, 1948 2	17 y/M	1	Headache	Craniotomy	Improved	N/A	N/A
2	Holland and Graham, 1958 17	31 y/F	1	Blurred vision Nausea Headache Weakness of right leg	Craniotomy	Dead	N/A	N/A
3	Amacher and Page, 1971 24	21 y/F	1	Headache Vomiting Nausea	Craniotomy + VC shunt	Improved	N/A	1 year
4	Yoshioka et al, 1985 25	Mean age = 35.3 y/M = 3	3	Gait disturbance Cerebellar ataxia	Craniotomy + shunt	Improved	N/A	1 year
5	Rifkinson-Mann et al, 1987 18	Mean age = 47 y/M = 2	2	Hemiparesis Hemianopsia Headache Vomiting Blurred vision	Craniotomy	Improved	N/A	1 year
6	Aesch et al, 1991 16	35 y/M	1	Ataxia Headache Nausea	VP shunt	Improved	N/A	2 months
7	Osaka et al, 1995 19	20 y/F	1	Headache Nausea Papilledema	Craniotomy	Improved	N/A	N/A
8	Hashish et al, 1999 26	Mean age = 51.5 y F = 2	2	Headache Gait disturbance Nausea Memory disturbance	Craniotomy + VC shunt	Improved	N/A	6 years
9	Suehiro et al, 2000 29	27 y/F	1	Dizziness Headache Nausea	ETV	Improved	N/A	N/A
10	Huang et al, 2001 20	15 y/F	1	Headache Nausea Vomiting Amenorrhea	Craniotomy	Improved	N/A	14 months
11	Carpentier et al, 2001 10	58 y/F	1	Visual impairment Dizziness Headache Nausea Vomiting Gait disturbance	ETV	Improved	N/A	3 years
12	Inamura et al, 2001	9 months/M	1	Macrocephaly Arrest of mental development	VP shunt	Improved	N/A	3 months
13	Karachi et al, 2003 11	Mean age = 47.3 y/M = 1, F = 2	3	Headache Vomiting Papilledema Vertigo Nausea Gait disturbance Sphincteric disorders Impairment of higher functions	ETV	Improved	N/A	36 months
14	Mohanty et al, 2008 1	>2 years	12	N/A (not specified in respect to PFVOO cases)	ETV	N/A (in respect to PFVOO cases)	Failed to describe (not specified in respect to PFVOO cases)	4.2 years
15	Longatti et al, 2009 4	60.6 years	10	Ideomotor slow down Gait disturbance Depression Dizziness Memory impairment Incontinence Visual impairment Headache Vomiting	ETV = 8 ETV + aqueductoplasty = 1 Endoscopic magendieplasty = 1	Improved = 9 Lost to follow-up = 1	N/A = 7 1. Recurrence after 12 years, re-ETV done 2. Recurrence after 3 years/ VP shunt done 3. Recurrence after 2 months, re-ETV done	47.7 months
16	Hashimoto et al, 2014	20 months/M	1	Syndrome of inappropriate antidiuretic hormone secretion	ETV	Improved	N/A	N/A
17	Torres-Corzo et al, 2014 6	18.5 years/M = 2, F = 5	7	Lethargy Bulging fontanel Headache Gait disturbance Seizures Blurring of vision Nausea Vomiting	ETV + magendieplasty = 5 ETV + magendieplasty + aqueductoplasty = 1 Endoscopic magendieplasty = 1	Improved	N/A	26.5 months
18	Ishi et al, 2015 15	3 y/M	1	Headache Vomiting	ETV	Improved	Recurrence after 1 year, re-ETV done	32 months
19	Kasapas et al, 2015 21	37 y/F	1	Headache Blurred vision Vomiting Phonophobia Recent memory loss Bilateral papilledema	Craniotomy	Improved	N/A	2 weeks
20	Duran et al, 2017 22	19 y/F	1	Headache Diplopia Intracranial hypertension	Craniotomy	Improved	N/A	N/A
21	Pérez et al, 2019	41 y/F	1	Headache Imbalance Nausea Vomiting	ETV	Improved	N/A	6 months
22	Bai et al, 2019 23	15 y	1	Headache Vomiting	Craniotomy	Improved	N/A	1 year
23	Rosa et al., 2021 14	7 y/M	1	Abdominal pain Vomiting Sixth and seventh cranial nerve palsy	VP Shunt twice VA Shunt once	Deteriorated	ETV after 10 months	6 years
24	Krejčí et al, 2021 7	Mean age = 40.9 y/M = 3, F = 5	8	Headache Vertigo Gait disturbance Diplopia Vomiting Papilledema	ETV = 5 Craniotomy = 2 Acute ventricular drainage = 1	Improved = 7 Death = 1	1. VP shunt in 1 patient 2. Recurrence after 6 weeks, re-ETV done	75.4 months
25	This study	50 y/F	1	Headache Difficulty in walking Imbalance while standing and walking Diplopia Vertigo Papilledema Three to four episodes of loss of consciousness	VP shunt	Improved	Not done	3 months
26	Summary	Mean age = 28 years/M = 17, F = 24 Sex N/A = 23	Total = 64 patients	Headache 50% ( *n* = 32) Vomiting 31.3% ( *n* = 20) Gait abnormalities 31.3% ( *n* = 20) Diplopia 6.25% ( *n* = 4) Vertigo 9.38% ( *n* = 6) Papilledema 12.5% ( *n* = 8) Cranial nerve palsy 1.6% ( *n* = 1) Raised intracranial pressure 3.1% ( *n* = 2) Memory loss = 6.25% ( *n* = 4) Seizure = 1.5% ( *n* = 1) Phonophobia = 1.5% ( *n* = 1) Loss of consciousness = 1.5% ( *n* = 1) Lethargy = 1.5% ( *n* = 1) Bulging fontanel = 1.5% ( *n* = 1) Vision abnormalities = 9.4% ( *n* = 6) Syndrome of inappropriate antidiuretic hormone secretion = 1.5% ( *n* = 1) Amenorrhea = 1.5% ( *n* = 1) Incontinence = 3.1% ( *n* = 2) Abdominal pain = 1.5% ( *n* = 1) Depression = 1.5% ( *n* = 1)	Craniotomy = 10 Shunt = 7 ETV = 23 Endoscopic magendieplasty = 8 Endoscopic aqueductoplasty = 2	Improved = 48 (75%) Deteriorated = 1 (1.5%) Death = 2 (3%) Lost to follow-up = 1 (1.5%) N/A = 12 (19%)	ETV = 5 (7.8%) Shunt = 2 (3.1%)	Mean follow-up = 20.9 months

Abbreviations: ETV, endoscopic third ventriculostomy; F, female; M, male; PFVOO, Primary fourth ventricle outlet obstruction; Ventriculo-cisternal shunt; VP, ventriculoperitoneal shunt; y, years.

## Discussion

### Etiopathogenesis and Demography

Tetraventricular hydrocephalus resulting from FVOO is caused due to multiple conditions. Hydrocephalus due to intracranial hemorrhage or intracranial infection is more common, occurring due to CSF malresorption, leading to lower endoscopic third ventriculostomy (ETV) success rates in these cases. 1 In IFVOO, hydrocephalus is principally obstructive in nature owing to the membranous occlusion of the foramina of Luschka and Magendie. The underlying mechanism leading to this occlusion is unknown. It may be congenital or acquired. 4 Congenital occlusion of the foramina of Luschka and Magendie is well-documented. 2 3 5 6 However, it fails to explain the exact pathogenesis leading to congenital cases of IFVOO becoming symptomatic. In acquired causes, the mechanism that has been suggested is underlying inflammation leading to scarring of the arachnoid layers in the cisterns and ventricles. This was supported by the findings of anomalous membranous proliferation in the interpeduncular cistern and thickening of the floor of the third ventricle. 7 In cases of ETV failure, signs of scarring in the interpeduncular cistern were found. 8 9 Multiple studies have shown an increased number and toughness of the membranes in the interpeduncular cistern, abnormal third ventricle floor rigidity, and changes in the choroidal plexus in the ventricle, leading to a higher risk of ETV failure in such cases. 1 4 6 The other explanation is that the increased intracranial pressure leads to a dilated fourth ventricle, which causes the membranes of foramina of Luschka and Magendie to come into contact with the dura mater, interrupting the CSF flow. 10 This might be the underlying mechanism in patients with acute hydrocephalus. However, there seems to be a lack of consensus regarding the primary cause. IFVOO is typically seen in adults, with no gender predilection. 7 The authors propose the fact that since the majority of the affected patients are adults, there appears to be an ongoing subclinical inflammatory process in the patients of IFVOO over a prolonged time period. This might lead to IFVOO with the congenital anomaly being present in the background.

### Clinical Presentation

Headache and gait difficulties are the most commonly encountered symptoms in IFVOO. Krejčí et al reported that primary surgery was successful in patients who had headaches in the preoperative period. 7 Symptoms of normal-pressure hydrocephalus were described in patients with obstructive tetraventricular hydrocephalus in the literature. 11 It becomes difficult to distinguish patients with normal pressure hydrocephalus (NPH) from IFVOO, as NPH is a more common entity.

### Radiologic Findings

Both IFVOO and communicating tetraventricular hydrocephalus present with Hakim's triad—progressive gait impairment, cognitive deficits, and urinary urgency and/or incontinence. It is difficult to distinguish them on clinical grounds. Brain MRI is the imaging modality of choice in such cases. Krejčí et al reported that the presence of ballooning of the fourth ventricle, decreased prepontine cistern volume, decreased retrocerebellar space, and concomitant anterior displacement of the brainstem were found to be specific for IFVOO. 7 However, these radiological features appear to be diagnostic of FVOO rather than IFVOO. As IFVOO leads to an obstructive type of hydrocephalus, preoperative MRI findings such as third ventricle bowing and concomitant fourth ventricle ballooning appear to indicate the diagnosis of IFVOO. Resolution of third ventricle bowing and fourth ventricle ballooning was associated with successful management of IFVOO. 7 12 13 The presence of a widely dilated aqueduct of Sylvius differentiates IFVOO from a trapped fourth ventricle. 4

## Treatment and Outcome

Treatment modalities for IFVOO include ETV, 13 14 15 shunt surgery, 3 16 open fenestration via suboccipital craniotomy, 2 17 18 19 20 21 22 23 or a combination of the two. 24 25 26

Suboccipital craniotomy and wide opening of the membrane were performed based on the fact that by removing the obstruction of the fourth ventricular outlets, CSF flow could be normalized. 17 24 Frequent recurrences of the hydrocephalus were seen, although the patients showed initial clinical improvement with ventricle sizes becoming near normal. 27 28

The use of ETV in FVOO was first described by Mohanty et al, where they reported an overall success rate of 65%, with favorable outcomes (91% success) in patients of age more than 2 years, with failure in all patients younger than 6 months of age. 1 15 Other studies have also reported successful outcomes using ETV for IFVOO, although in one study, redo ETV was done following recurrence of hydrocephalus. 15 29

In the literature, the majority of patients with IFVOO underwent endoscopic intervention. In patients with IFVOO, ETV failure can occur at any time, in comparison to the other types of hydrocephalus, in which failure typically occurs in the first few weeks. 4 30 Literature review has found that in cases of IFVOO, ETV has a higher failure rate compared with the rest of the treatment options. 7

Some cases of IFVOO present with a substantial increase in the volume of the fourth ventricle, which may lead to small prepontine and suprasellar cisterns due to the brainstem being pushed anteriorly. This may lead to the basilar artery being pushed closer to the third ventricle floor, making ETV an unsafe procedure. In such cases, Endoscopic fourth ventriculostomy as described by Giannetti et al can be performed. ETV has its own advantages as it is safer to perform. In endoscopic fourth ventriculostomy, there may occur extra manipulation of the third ventricle, cerebral aqueduct, and fourth ventricle with the increased risk of damage to the surrounding neurovascular structures. Hence, it should be considered a surgical option only in cases when ETV is not possible. 31

Ventriculoperitoneal shunts have been utilized for the management of IFVOO, although long-term follow-up is not available in such cases. 3 16 Hence, it is difficult to assess the efficacy of ventriculoperitoneal shunts in such cases.

## Conclusion

The precise mechanism by which IFVOO develops and causes tetraventricular hydrocephalus is still unknown. Obstructive hydrocephalus results from IFVOO; hence, ETV is considered to be the treatment of choice in such cases. But in such cases, the risk of ETV failure remains higher. Literature review suggests long-term follow-up in patients undergoing ETV, as the failure can occur at any time. The initial treatment procedures, such as open fenestration via craniotomy and shunt surgery, remain still relevant and effective procedures for IFVOO. An alternate line of treatment for it would be endoscopic fenestration of the fourth ventricle outlets. Larger studies involving multiple hospitals are required to confirm these findings.
